# The effect of a mindful mothering nursing intervention virtual program on mothers with adverse childhood experiences: a randomized controlled trial

**DOI:** 10.3389/fpubh.2025.1599604

**Published:** 2025-06-11

**Authors:** Eunjeong Cho, Gisoo Shin

**Affiliations:** ^1^Department of College of Nursing, Hallym Polytechnic University, Chuncheon, Republic of Korea; ^2^College of Nursing, Chung-Ang University, Seoul, Republic of Korea

**Keywords:** adverse, childhood, experiences, mothering, mindfulness, intervention

## Abstract

**Background:**

Mothers who have experienced adverse childhood experiences often exhibit negative parenting attitudes and behaviors.

**Objectives:**

The study we are submitting for consideration is a randomized controlled trial that developed and implemented a mindful mothering nursing intervention virtual program for mothers who have experienced adverse childhood experiences. The primary aim of the study was to evaluate the effectiveness of this intervention.

**Subjects:**

The participants of this study comprised 60 mothers who were raising children aged 3 to 7 and had experienced adverse childhood experiences before the age of 18. The participants were randomly assigned to either the experimental group or the control group.

**Tools:**

The mindful mothering nursing intervention virtual program was administered to the experimental group over a period of 6 weeks, from June 20 to July 29, 2022. The intervention consisted of weekly sessions, each lasting 120 min, for a total of six sessions. The program covered a range of topics including self-understanding, awareness of adverse childhood experiences, mindfulness based parenting and emotional regulation techniques, understanding children’s physical and emotional developmental stages and corresponding parenting attitudes, emotion coaching and child acceptance, strategies for utilizing social support, professional counseling, and self-reflection. To evaluate the effectiveness of the intervention, validated instruments were used to measure adverse childhood experiences, parenting stress, parenting efficacy, mindful parenting attitude, perceived social support, and parenting behavior. Data collection was conducted both prior to the intervention and again 48 h after its completion.

**Result:**

In this study, the experimental group showed a statistically significant reduction in mean scores for parenting stress, an increase in parenting efficacy, an improvement in mindful parenting attitude, and more positive parenting behavior compared to the control group. However, there was no statistically significant difference between the two groups in perceived social support (*p* < 0.05).

**Implications:**

This underscores the importance of actively implementing parenting nursing interventions for mothers who have experienced adverse childhood experiences to prevent the intergenerational transmission of adverse childhood experiences.

**Conclusion:**

Based on the findings of this study, the development of various parenting nursing intervention programs to support mothers with adverse childhood experiences is essential.

**Clinical trial registration:**

https://cris.nih.go.kr/cris/search/detailSearch.do?seq=21959&search_page=L, identifier KCT 0007353.

## Background

According to global statistics from the year 2024, approximately four hundred million children under the age of five, which accounts for about 60 % of that age group, are regularly subjected to psychological violence or physical punishment. Furthermore, it is estimated that around six hundred and fifty million women and girls worldwide have experienced sexual violence before reaching the age of 18, as reported by the United Nations Children’s Fund (1). In South Korea, data reported in the year 2023 indicate that the incidence of child abuse reached three hundred sixty-four point one cases per one hundred thousand children, reflecting a high prevalence. Among these cases, approximately 85 % occurred within the family setting. Particularly concerning is the early detection rate of child abuse in South Korea, which stands at only three point six 4 %. This rate is significantly lower than that of other member countries of the OECD, such as the United States at seven point 7 % and Australia at 11 point 7 % (2).

Child abuse is categorized as an adverse childhood experience (ACE), which encompasses traumatic stress resulting from physical, emotional, and sexual abuse, neglect, and exposure to domestic violence among children under the age of 18 (3–6). ACE has been shown to negatively impact interpersonal relationships due to the development of insecure attachment from childhood, and can impair self-regulation abilities (7). Repeated exposure to ACE excessively stimulates the limbic system and disrupts the normal regulation of the hypothalamic–pituitary–adrenal axis, leading to adverse effects on brain development (4–6).

Most critically, individuals who have experienced ACE are at an increased risk of perpetuating abuse as parents (6, 8, 9). This is attributed to the strong correlation between ACE and parenting stress, where the experience of childhood adversity contributes to intergenerational transmission of abuse. The effects are particularly pronounced in mothers who have experienced ACE, as they are more likely to engage in neglectful or punitive parenting, display lower levels of empathy, and exhibit inconsistent caregiving behaviors (10). These parenting practices not only lead to issues such as inattention, hyperactivity, impulsiveness, and aggression in children but also contribute to psychological and emotional problems, including anxiety and depression (5, 6, 9).

Furthermore, these issues extend into the school environment, increasing the likelihood of peer violence and, in adulthood, escalating to dating and domestic violence (6). Recognizing the far-reaching consequences of ACE, the WHO has designated addressing the lifelong physical, mental, and social impairments caused by ACE as a critical public health priority (3).

Structured parenting intervention programs have been shown to positively influence parenting attitudes among mothers who have ACE. These programs assist mothers in improving their emotional regulation, enhancing empathy, and acquiring consistent parenting strategies (5, 6). As a result, punitive parenting behaviors rooted in past experiences of abuse are reduced, enabling mothers to respond more sensitively to their children’s needs and to engage in more stable and supportive parenting practices (11). Such improvements play a critical role in preventing the intergenerational transmission of abuse (4–6, 10).

Mindful parenting, in particular, is a parenting approach in which the mother intentionally brings her full attention to the present moment while interacting with her child. This approach involves accepting emotions and thoughts without judgment and responding sensitively to the child’s needs and emotional signals (12, 13). Mindful parenting applies the core principles of traditional mindfulness to the parenting context, fostering mothers’ emotional regulation and self-awareness (14). It also helps reduce stress and reactive behaviors, thereby promoting more positive and nurturing relationships with their children (12–15). This approach is especially effective for mothers with a history of child abuse, as it supports the restoration of emotional regulation and empathy (6, 16).

Meanwhile, social cognitive theory explains that human behavior is shaped by the dynamic interaction among personal factors, behaviors, and environmental influences. This theoretical framework is valuable for understanding the psychological and behavioral variables related to parenting (17). Parenting stress refers to the emotional strain and sense of burden experienced by parents in the process of raising children. When persistent, parenting stress can lead to increased aggressive or inconsistent negative parenting behaviors. In contrast, parenting efficacy refers to a mother’s belief in her ability to parent effectively. Mothers with high parenting efficacy tend to adopt more consistent and stable parenting practices, even in challenging situations (18, 19).

A mindful parenting attitude, defined as a parent’s effort to stay emotionally connected with the child while remaining attentive to the present moment, enhances self-awareness and emotional regulation. This, in turn, reduces stress and contributes to improved parenting efficacy (13, 14, 16). Additionally, perceived social support, or a mother’s belief that she receives emotional and instrumental assistance from others, acts as a protective buffer during stressful situations and promotes positive parenting behaviors (9, 11). From the perspective of social cognitive theory, parenting stress may have a detrimental effect on parenting behaviors. However, internal and external resources such as parenting efficacy, a mindful attitude, and social support can buffer these negative effects and foster healthier parenting practices (17, 19).

Based on social cognitive theory, the present study aimed to develop and evaluate a parenting intervention program incorporating mindful parenting for mothers with a history of ACE.

### Theoretical background

Women who have experienced adverse events in childhood, such as abuse, neglect, or emotional deprivation, are more likely to encounter psychological and emotional difficulties during the transition to motherhood (5, 8, 10). These early experiences can lead to diminished self-esteem, challenges in forming secure attachments, anxiety related to parenting, and difficulties in emotional regulation (11). Such issues may disrupt healthy interactions with their children and contribute to the development of maladaptive parenting practices (6, 8). In particular, negative parenting experiences from the past may be unconsciously reenacted or lead to excessive efforts to avoid such behaviors, resulting in confusion and stress regarding the maternal role (5, 8, 10). These unresolved emotional wounds can distort perceptions of current parenting situations and hinder the establishment of a coherent maternal identity (18). Therefore, mothers with such histories require professional psychosocial support and therapeutic intervention, both of which are essential for the restoration of healthy maternal functioning and for the developmental well-being of their children (6, 11).

Parenting intervention programs grounded in social cognitive theory have evolved to address broader issues related to generalizability and mechanisms of behavioral change (12, 15). These programs are increasingly examined for their potential to explain the mechanisms through which positive changes in parenting attitudes and behaviors occur among mothers with adverse childhood experiences (4, 5, 8, 10). According to social cognitive theory, parenting behaviors can be shaped through direct and indirect modeling, whereby exposure to positive parenting examples leads to the adoption of healthier practices (17). This modeling process can take various forms and includes reinforcement, allowing learned behaviors to be maintained across different contexts and over time (19). As such, interventions targeting the parenting attitudes and behaviors of mothers with early adverse experiences have focused on improving parenting quality as a means of preventing intergenerational transmission of abuse (5, 6, 11).

Notable examples of evidence-based parenting programs include the ACT raising safe kids program, the Triple P positive parenting program, and the incredible years program (20). The ACT program, developed by the American Psychological Association, is designed to prevent child maltreatment and promote positive parenting skills among parents and caregivers of children from birth to age 10. It addresses topics such as nonviolent discipline, child development, anger management, conflict resolution without violence, the impact of violent media on children, and strategies for child protection (21). The Triple P program provides parents with tailored knowledge and skills to build confidence and promote positive parenting practices. Similarly, the Incredible Years program, designed for parents of children aged 4 to 12, helps prevent and manage behavioral problems in children effectively (20, 21).

Mindfulness is defined as the intentional, nonjudgmental awareness of the present moment. It fosters self-acceptance and reduces internal conflict through cognitive awareness, thereby positively influencing parenting attitudes. Mindfulness-based parenting has been shown to improve consistency and flexibility in parenting approaches and to reduce automatic negative responses toward children. Core components of mindful parenting include attentive listening, nonjudgmental acceptance of both self and child, emotional awareness, self-regulation, and self-compassion (12, 14, 16). Previous research has demonstrated that mindful parenting effectively reduces parenting stress and enhances parent–child relationships (16).

The distinction between social learning and mindfulness lies more in their conceptual foundations than in empirical practice. However, the potential integration of these models has been highlighted in meta-analyses of mindful parenting interventions. These analyses have shown that the most favorable outcomes in parenting behavior are associated with studies that employed specific behavioral techniques, suggesting that the mechanisms of change may reside more in applied methods than in conceptual frameworks alone (22, 23).

In this study, we took a novel approach by developing the mindful mothering nursing intervention virtual program (MMNI), which uniquely integrates social cognitive theory with a mindful parenting framework. This integration was purposefully designed to offer a more comprehensive and theoretically grounded strategy for supporting mothers with a history of ACE, thereby addressing a critical gap left by previous intervention studies.

### The study objectives

This study aimed to develop the MMNI for Korean mothers with ACE and evaluate its effectiveness using a randomized control-group pretest-posttest design. To develop the program, a comprehensive review of previous studies on child maltreatment and parenting programs was conducted, analyzing their findings. Based on this review, in-depth interviews were conducted with 10 mothers who had experienced ACE to assess their needs for the program. Subsequently, recent parenting programs implemented overseas were analyzed, with reference to evidence-based resources such as the California evidence-based clearinghouse for child welfare (CEBC) and national registry of evidence-based programs and practices (NREPP), which aligned most closely with the objectives of this study.

To ensure the validity of the topics and content in MMNI of this study, expert consultations were conducted. The final program was structured as a virtual intervention consisting of six sessions, totaling 12 h. Each session lasted 120 min and was facilitated by the researcher. Additionally, mindful meditation exercises and an open chatroom were provided to allow participants to engage with the program beyond scheduled sessions, ensuring accessibility regardless of time and location.

To evaluate the intervention effects of the MMNI, the study formulated the following hypotheses:

*H1*: The experimental group will experience a greater reduction in parenting stress compared to the control group after program participation.

*H2*: The experimental group will show a greater increase in parenting efficacy compared to the control group after program participation.

*H3*: The experimental group will demonstrate a greater improvement in mindful parenting attitudes compared to the control group after program participation.

*H4*: The experimental group will perceive a greater increase in perceived social support compared to the control group after program participation.

*H5*: The experimental group will exhibit a greater increase in positive parenting behaviors compared to the control group after program participation.

## Methodology and materials

### Study design

**Figure 1:**
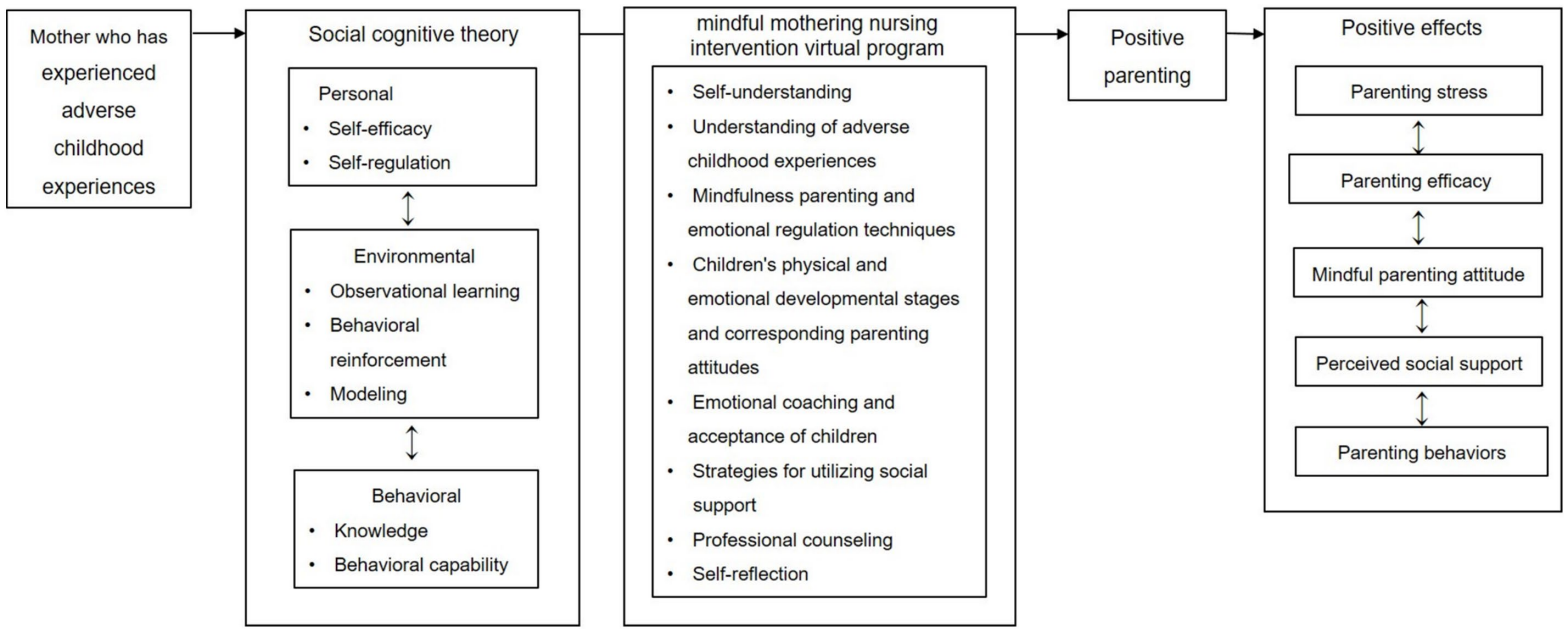
Conceptual framework of this study.

**Figure 2:**
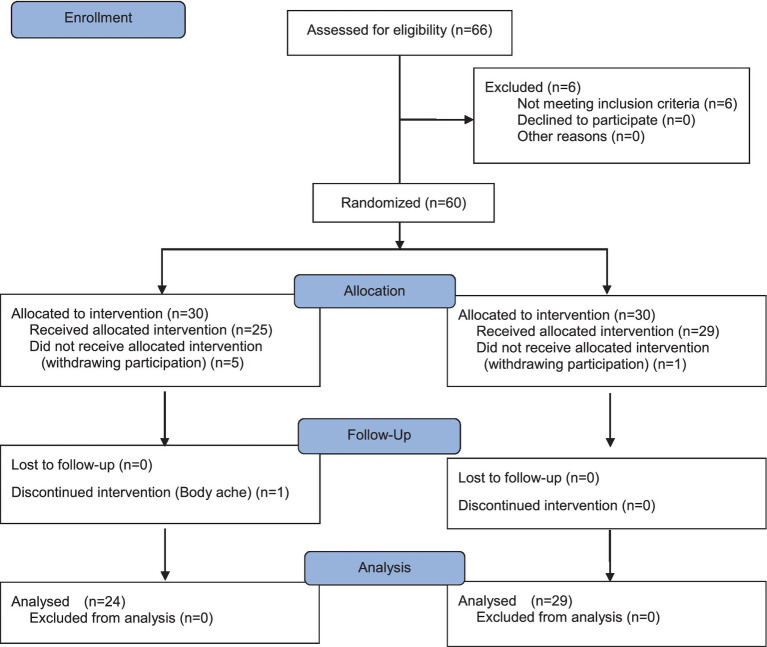
Consort flow diagram for individual randomized controlled trial of the intervention.

This study employed a randomized control-group pretest-posttest design to develop and evaluate the effectiveness of the MMNI for Korean mothers with ACE. The conceptual framework an flowchart of study are illustrated in Figures 1, 2.

### Setting and participants

The participants were mothers residing in two cities in South Korea who were raising children aged 3 to 7 years and had experienced ACE before the age of 18. Mothers with physical or psychological difficulties, communication disorders, or those currently taking anxiolytics or sedatives were excluded from the study.

To recruit participants, advertisements were posted in local kindergartens and social network communities within the Seoul and Gyeonggi, South Korea. Mothers who expressed interest in participating were provided with a detailed explanation of the study’s objectives and procedures. Those who voluntarily provided written informed consent were then asked to complete a screening survey to determine their ACE history. The ACE questionnaire is generally interpreted as follows: scores of 3 or lower indicate relatively low risk; scores of 4 or higher are associated with a significantly increased risk of mental health issues, physical health problems, and behavioral difficulties; and scores of 6 or above are considered indicative of a high-risk group for outcomes such as suicide attempts, substance abuse, and chronic illnesses (24). In this study, participants with ACE scores of 4 or higher were included. Only those who met the eligibility criteria were randomly assigned to either the experimental or control group. Participants who were currently taking psychiatric medication due to past ACE were excluded from this study. However, it was clearly explained to all participants that they could withdraw from the study at any time if they experienced physical or psychological discomfort related to past ACE during the course of the intervention. In such cases, they would be referred to a licensed counselor or medical professional for appropriate support.

### Sample size

The required sample size was determined using G*Power 3.1.9.2 software. Based on previous studies on parenting intervention programs for child maltreatment prevention (25), the sample size was calculated with a significance level (*α*) of 0.05, statistical power (1-*β*) of 0.80, and an effect size of 0.8. The results indicated that 26 participants per group were required, totaling 52 participants. Considering potential attrition, the sample size was increased to 60 participants (30 in the experimental group and 30 in the control group).

### Random assignment

A total of 60 mothers who met the eligibility criteria were assigned a unique identification number based on the order of their study registration. The plan was to allocate 30 participants to the experimental group and 30 to the control group. To ensure randomization, a computerized randomization tool was used to generate randomized allocation numbers, which were then used to automatically assign participants to either the experimental or control group. Before the intervention, all 60 participants completed the pretest survey. However, five participants in the experimental group withdrew from the study due to concerns about potential identification by others, and one participant in the control group was lost to follow-up. As a result, the final sample consisted of 25 participants in the experimental group and 29 in the control group. To maintain single blinding in this randomized controlled trial, participants were not informed of the specific details of the intervention program and were unaware of whether they had been assigned to the experimental or control group.

### Measurements

#### Adverse childhood experiences

ACE were assessed using the Korean-translated version of the ACE questionnaire for adults – identified, originally developed by Felitti et al. (24). This instrument comprises 10 items, each requiring a binary response (‘Yes’ or ‘No’). A higher number of ‘Yes’ responses indicates a greater severity of ACE. In this study, the reliability of the scale was Cronbach’s *α* = 0.87.

#### Parenting stress

Parenting stress was measured using the Korean-translated version of the parenting stress index short form (PSI-4-SF) and translated by Jeong et al. (26). This instrument consists of 36 items, categorized into three subscales: Parental distress (12 items), dysfunctional parent–child interaction (12 items), and difficult child (12 items). Each item is rated on a 5-point Likert scale ranging from 1 (strongly disagree) to 5 (strongly agree), with higher scores indicating greater parenting stress. The reliability of the instrument in this study was Cronbach’s *α* = 0.95 for both pretest and posttest.

#### Parenting efficacy

Parenting efficacy was assessed using the Korean-translated version of the tool to measure parenting self-efficacy. The translation was conducted following the guidelines provided by the WHO (27), and its content validity was verified through expert consultation (three nursing professors, two pediatricians, one pediatric nurse specialist, and one psychiatric nurse specialist). The instrument consists of 48 items across eight subdomains and each item is rated on an 11-point Likert scale from 0 (strongly disagree) to 10 (strongly agree), with higher scores indicating greater parenting efficacy. The reliability of this instrument in this study was Cronbach’s *α* = 0.97 for both pretest and posttest.

#### Mindful parenting attitude

Mindful parenting attitude was measured using the Korean-translated version of the interpersonal mindfulness in parenting scale, translated by Kim et al. (13). This instrument consists of 18 items and each item is rated on a 5-point Likert scale from 1 (strongly disagree) to 5 (strongly agree), with higher scores indicating a higher level of mindful parenting. The overall reliability of the instrument in this study was Cronbach’s *α* = 0.86 (pretest) and Cronbach’s *α* = 0.87 (posttest).

#### Perceived social support

Perceived social support was measured using the instrument developed by Park (28). This instrument consists of 25 items and each item is rated on a 5-point Likert scale from 1 (strongly disagree) to 5 (strongly agree), with higher scores indicating a higher level of perceived social support. The reliability of the instrument in this study was Cronbach’s *α* = 0.97 (pretest) and Cronbach’s *α* = 0.98 (posttest).

#### Parenting behavior

Parenting behavior was assessed using the Korean-translated version of the instrument developed by Silva (29). This instrument consists of 30 items, and the subdomains of parenting style and parental behavior are rated on a 5-point Likert scale, and the media discipline item is rated on a 4-point Likert scale. A higher score indicates more positive parenting behavior. The reliability of the instrument in this study was Cronbach’s *α* = 0.89 for both pretest and posttest.

## Data collection and analysis

### Pretest

The pretest was conducted from June 13 to June 19, 2022, with 60 participants who agreed to participate in the study. Prior to the pretest, an orientation session was held from June 7 to June 10, 2022. The pretest was conducted online via a questionnaire covering demographic characteristics, parenting stress, parenting efficacy, mindful parenting attitude, perceived social support, and parenting behavior.

### Experimental treatment

Following the orientation, the MMNI, designed for mothers with children aged 3 to 7, was conducted via Zoom. Participants assigned to the experimental group were provided with an individual URL to access the program, while the control group received a parenting education manual from the Korea healthy family promotion institute via text message. The MMNI was administered to the experimental group over a period of 6 weeks, from June 20 to July 29, 2022. The intervention consisted of weekly sessions, each lasting 120 min, for a total of six sessions. In the first week, participants engaged in a life graph drawing activity designed to promote self-understanding and awareness of their ACE. In the second week, the focus was on acquiring knowledge related to stress, event-related trauma, and developmental trauma stemming from childhood ACE. Participants also practiced breathing meditation techniques aimed at regulating internalized negative emotions. During the third week, participants explored the stages of physical and emotional development in children and learned emotion regulation strategies modeled to enhance self-efficacy and foster a more accepting parenting attitude through self-regulation. The fourth week emphasized the use of social support strategies, including emotional coaching through professional counseling. Participants were also guided through self-reflection exercises. In the fifth week, role-playing activities were conducted based on various scenarios, including loving-kindness meditation and communication skills with children. The sixth week provided an opportunity for participants to review their relationships with their children, reflect on broader social relationships with family and friends, and receive information about professional support systems available within the community. Throughout the 6-week program, participants received regular text messages encouraging the practice of walking meditation and breathing exercises for emotional self-regulation. They were asked to respond regarding their participation and, before sleep or during parent–child interactions, to express their emotions metaphorically using colors and report them to the researcher. In addition, an alert system was made available throughout the intervention period for participants to request support when needed, and referrals to professional services were arranged as necessary.

The content provided to the control group consisted of general educational materials commonly offered to mothers and was entirely different from the intervention content received by the experimental group. For example, the control group materials addressed topics such as normal physical development in children, vaccination schedules and procedures, safety management, hygiene practices, and nutritional care. One participant in the experimental group withdrew from the study before completing the program; accordingly, all pre-intervention survey data from this participant were discarded. As the program was conducted virtually, the URL for the intervention was provided exclusively to participants in the experimental group, making face-to-face interaction or contact information exchange between the experimental and control groups impossible. Participants in the experimental group were granted unlimited access to mindfulness resources, including breathing and walking meditation exercises. Additionally, they were encouraged to form a supportive community by participating in an open group chat with other members of the experimental group.

### Posttest

The posttest was conducted 48 h after the experimental treatment concluded, using the same questionnaire as the pretest. The posttest period was from August 1 to August 5, 2022, with 24 participants from the experimental group and 29 participants from the control group completing the survey. In addition, 7 days after the completion of the post-intervention survey, an informational email regarding the program provided to the experimental group was sent to the control group. For those who expressed interest, the same program was offered 1 month after its completion in the experimental group.

### Researcher preparation

To ensure the necessary qualifications for conducting this study, the researcher undertook several professional development efforts to acquire relevant knowledge and skills. The researcher completed *the ACT raising safe kids facilitator training workshop,* a parenting program developed by the American Psychological Association, which provided the foundation for fulfilling both counseling and facilitation roles during the mindfulness-based intervention. Additionally, the researcher completed a 40-h m*indfulness-based stress reduction* course, thereby gaining the competency to lead mindfulness meditation practices and serve as an educator for mothers with adverse childhood experiences. Furthermore, the researcher received training in focus group interview methodology, which enabled the use of in-depth interviewing techniques to elicit participants’ deeper insights and experiences.

### Data analysis

Data analysis was conducted using SPSS 26.0 (IBM Corp., Armonk, NY, USA). Descriptive statistics were used to analyze participants’ demographic characteristics. Baseline equivalence between the experimental and control groups was tested using independent t-tests for continuous variables and Chi-square tests or Fisher’s exact tests for categorical variables. Skewness and kurtosis were assessed to check the normality of key variables. Pretest-posttest differences in parenting stress, parenting efficacy, mindful parenting attitude, perceived social support, and parenting behavior within each group were analyzed using paired sample t-tests. Differences in post-intervention mean scores between the experimental and control groups were analyzed using independent t-tests. Also, The effect size of the program was calculated using Cohen’s *d*, which is obtained by dividing the difference in means between the two groups by the pooled standard deviation. According to Cohen’s criteria, a *d* value of 0.2 or less was interpreted as a small effect size, 0.5 as a medium effect size, and 0.8 or greater as a large effect size (30).

### Ethical considerations

This study was approved by the institutional review board of the university to which the researcher is affiliated on April 20, 2022 (No: 1041078-202112-HR-345-01), and received approval as a registered clinical research institution from the national institutes of health and the Korea disease control and prevention on May 3, 2022. (No: KCT 0007353)^1^.

Prior to the study, participants were fully informed about the purpose of the research, the confidentiality of the data, and the data disposal procedures after the conclusion of the study. Consent for voluntary participation was obtained. Additionally, participants were informed that they could withdraw from the study at any time without any negative consequences. Furthermore, all measurement tools used in this study were utilized only after receiving permission from the original authors and the translators.

## Results

### General characteristics and homogeneity verification of participants

The average age of the participants was 34.63 ± 7.77 years in the experimental group and 34.14 ± 7.24 years in the control group. Regarding marital status, 23 participants (95.8%) in the experimental group and 27 participants (93.1%) in the control group were married. In terms of education level, the highest proportion of participants in both groups were university graduates, accounting for 66.7% in the experimental group and 72.4% in the control group. Additionally, the most common number of children aged 3 to 7 years was two, reported by 58.3% of the experimental group and 51.7% of the control group.

There were no statistically significant differences between the two groups in terms of average age, religion, marital status, education level, employment status, number of children, average monthly income, confirming the pre-experimental homogeneity of the groups (Table 1).

**Table 1:** Baseline characteristics and homogeneity testing of participants.

(*N*=53)						
Variables	Categories	Exp. Group (*N* = 24)	Cont. Group (*N* = 29)	Total (*N* = 53)	*χ*^2^ / *t*	*p*
		*N* (%) or Mean±SD	M (%) or Mean±SD	M (%) or Mean±SD		
Age (years)		34.63 ± 7.77	34.14 ± 7.24	34.36 ± 7.41	0.24	0.814
Religion	Christian	8 (33.3)	6 (20.7)	14 (26.4)	2.25	0.523
	Buddhism	3 (12.5)	2 (6.9)	5 (9.4)		
	Catholic	2 (8.3)	5 (17.2)	7 (13.2)		
	No Religion	11 (45.8)	16 (55.2)	27 (50.9)		
Marital status	Married	23 (95.8)	27 (93.1)	50 (94.3)	0.18	0.669
	Unmarried	1 (4.2)	2 (6.9)	3 (5.7)		
Education level	High School	6 (25.0)	6 (20.7)	12 (22.6)	0.21	0.902
	College	16 (66.7)	21 (72.4)	37 (69.8)		
	Graduate School	2 (8.3)	2 (6.9)	4 (7.5)		
Occupation	No	18 (75.0)	21 (72.4)	39 (73.6)	6.15	0.631
	Yes	6 (25.0)	8 (27.6)	14 (26.4)		
Number of children	1	7 (29.2)	11 (37.9)	18 (34.0)	0.46	0.796
	2	14 (58.3)	15 (51.7)	29 (54.7)		
	3	3 (12.5)	3 (10.3)	6 (11.3)		
Monthly income (million won)		4.96 ± 2.21	5.42 ± 1.82	5.21 ± 2.00	0.84	0.407

SD, Standard deviation; ACEs, Adverse childhood experience; Exp, Experimental; Cont, Control.

### Homogeneity test of outcome variables before the intervention

There were no statistically significant differences between the experimental and control groups in the pre-experimental mean scores for parenting stress (*t* = 0.07, *p* = 0.291), parenting efficacy (*t* = −0.90, *p* = 0.372), mindful parenting attitude (*t* = −0.26, *p* = 0.799), perceived social support (*t* = −0.22, *p* = 0.829), or parenting behavior (*t* = −0.84, *p* = 0.404). These results confirm the pre-experimental homogeneity of the two groups (Table 2).

**Table 2:** Homogeneity test of outcome variables before the intervention.

(*N*=53)				
Variables	Exp. Group (*N* = 24)	Cont. Group (*N* = 29)	*t*	*p*
	Mean±SD	Mean±SD		
Parenting stress	3.08 ± 0.74	2.88 ± 0.67	1.07	0.291
Parenting efficacy	6.29 ± 1.64	6.69 ± 1.58	−0.90	0.372
Mindfulness parenting attitude	2.89 ± 0.65	2.94 ± 0.67	−0.26	0.799
Perceived social support	3.31 ± 0.89	3.36 ± 0.92	−0.22	0.829
Parenting behavior	3.13 ± 0.55	3.26 ± 0.51	−0.84	0.404

SD, Standard deviation; Exp, Experimental; Cont, Control.

### Hypothesis testing

The results of the hypothesis testing are presented in Table 3.

**Table 3:** Comparison of parenting-related outcomes before and after the intervention.

(*N*=53)							
Variables	Group	Pre Mean±SD	Post Mean±SD	Difference Mean±SD	*t*	*p*	Cohen’s *d*
Parenting stress	Exp.	3.08 ± 0.74	2.25 ± 0.49	−0.83 ± 0.55	−7.36	< 0.001	2.14
	Cont.	2.88 ± 0.67	2.90 ± 0.64	0.02 ± 0.12	0.92	0.365	
Parenting efficacy	Exp.	6.29 ± 1.64	7.45 ± 1.16	1.16 ± 1.15	4.92	< 0.001	1.26
	Cont.	6.69 ± 1.58	6.74 ± 1.47	0.06 ± 0.46	0.65	0.522	
Mindful parenting attitude	Exp.	2.89 ± 0.65	3.32 ± 0.66	0.43 ± 0.60	3.55	0.002	0.81
	Cont.	2.94 ± 0.67	2.99 ± 0.62	0.05 ± 0.29	1.00	0.327	
Perceived social support	Exp.	3.31 ± 0.89	3.75 ± 1.02	0.44 ± 0.84	2.56	0.018	0.53
	Cont.	3.36 ± 0.92	3.46 ± 0.85	0.10 ± 0.34	1.56	0.130	
Parenting behavior	Exp.	3.13 ± 0.55	3.49 ± 0.53	0.36 ± 0.48	3.66	0.001	1.17
	Cont.	3.26 ± 0.51	3.18 ± 0.52	−0.08 ± 0.23	−1.87	0.072	

SD, Standard deviation; Exp, Experimental; Cont, Control.

After the experimental treatment, the mean pre-post score for parenting stress decreased in the experimental group (−0.83 ± 0.55), whereas it slightly increased in the control group (0.02 ± 0.12). This supports Hypothesis 1 (*t* = −7.40, *p* = <0.001).

The mean pre-post score for parenting efficacy increased in both the experimental group (1.16 ± 1.15) and the control group (0.06 ± 0.46). However, the difference between the two groups was statistically significant, supporting Hypothesis 2 (*t* = 4.41, *p* = <0.001).

Similarly, the mean pre-post score for mindful parenting attitude increased in both the experimental group (0.43 ± 0.60) and the control group (0.05 ± 0.29), with a statistically significant difference between the two groups. This supports Hypothesis 3 (*t* = 2.86, *p* = 0.007).

Hypothesis 4, which proposed that perceived social support would increase more in the experimental group than in the control group, was rejected due to the absence of a statistically significant difference between the two groups (*t* = 1.87, *p* = 0.072).

Regarding positive parenting behavior, the mean pre-post score increased in the experimental group (0.36 ± 0.48) but decreased in the control group (−0.08 ± 0.23). This supports Hypothesis 5 (*t* = 4.10, *p* < 0.001).

## Discussion

This study was conducted to develop an MMNI based on social cognitive theory for mothers with ACE, and to verify its effectiveness. In this regard, the results will be discussed based on the hypotheses proposed in this study.

As a result of the study, Hypothesis 1 was supported, as the parenting stress of the experimental group participating in the MMNI was statistically significantly reduced compared to the control group. Parenting stress is experienced by all parents in general; however, for mothers who have experienced ACE, the psychological pain formed since childhood negatively impacts parenting behaviors, which in turn exacerbates parenting stress (4, 8, 10). In particular, these mothers experience difficulty in regulating their emotions in the context of intensified parenting stress, which can lead to child abuse (11, 18). Furthermore, through coping mechanisms such as neglect, harsh discipline, inconsistent parenting, and lack of empathy, ACE can contribute to an intergenerational cycle of adversity (4, 11). Given the detrimental effects of ACE on the mental health of both mothers and their children, addressing trauma-based stress is a critical public health priority (29). Parenting intervention programs that target the mental health of mothers with ACE can lead to positive improvements in parenting outcomes. A meta-analysis of group-based interventions demonstrated that such programs not only reduce stress among mothers with ACE but also have beneficial effects on parenting behaviors and perceived parenting efficacy (6). The MMNI in this study also incorporated self-understanding as the first step in the intervention, allowing participants to write their life graphs and share experiences to help them regulate their emotions objectively. Additionally, the program included techniques such as breathing meditation, loving-kindness meditation, and walking meditation to help participants regulate intense emotions. This can be seen as an intervention strategy emphasized in mindfulness-based parenting, where mothers are encouraged to shift their attention from various stresses in the parenting process toward themselves, and to accept their parenting environment as it is (7, 12). This self-regulation process interacts with self-efficacy and positively influences the reduction of parenting stress (9, 14).

Self-efficacy is also closely related to parenting efficacy (15–17), and in this study, the experimental group’s parenting efficacy showed a statistically significant difference compared to the control group, supporting Hypothesis 2. Parenting efficacy refers to a parent’s belief in their ability to effectively parent their child. Parents with high parenting efficacy tend to solve problems positively when unexpected difficulties arise in the parenting process (22). Moreover, parents with high parenting efficacy exhibit consistent parenting attitudes, which are crucial for the child’s physical development and emotional stability (25). In particular, the parenting efficacy of mothers with ACE provides the possibility of breaking the intergenerational transmission of ACE by helping them break free from negative past experiences (8, 11). Previous research has suggested child emotion coaching as one of the effective interventions to strengthen parenting efficacy, particularly for mothers with ACE (31). Child emotion coaching is a parenting method that helps mothers understand, empathize with, and appropriately express their child’s emotions (25). It not only addresses the mother’s emotions but also provides correct knowledge about child development at different stages and behaviors, as well as how to respect the child’s emotions (5, 10). Through this process, mothers are equipped with the ability to effectively understand and raise their children, which helps them gain confidence in parenting (4, 7). The MMNI in this study also included child emotion coaching, where role-playing based on various scenarios was used to help mothers understand and empathize with their children. This applied the behavioral determinants (knowledge, behavioral capability) proposed in social cognitive theory, strengthening non-violent parenting behaviors and training mothers to look into their children’s emotions (23, 25). Although ACE are often associated with an intergenerational cycle of adversity, many mothers demonstrate remarkable resilience in the face of such experiences. This resilience is frequently rooted in a strong desire to improve their children’s lives and a sense of parenting efficacy that motivates them to reconsider and transform traditional caregiving practices. These findings highlight the multidimensional and dynamic nature of intergenerational ACE transmission, underscoring the need for accessible, trauma-informed parenting interventions that build upon mothers’ inherent wisdom and strengths (22, 32).

In the results of this study, the experimental group’s mindfulness parenting attitude was statistically significantly higher than the control group, supporting Hypothesis 3. Mindfulness parenting attitude refers to the attitude of a parent who regulates emotions such as anger and frustration, and objectifies the current situation to accept the child’s emotions and behaviors during parenting (22). This interacts with parenting stress and parenting efficacy and is a crucial factor for mothers with ACE to practice open acceptance in their relationship with their children (12, 17). From the perspective of social cognitive theory, mindfulness parenting attitude strengthens mothers’ cognitive and emotional self-regulation abilities, creating a modeling process for parenting happiness (12, 33). To achieve this, it is important to provide correct knowledge about the difference between punishment and discipline, and to offer solutions to resolve mother–child conflicts (16, 20). Therefore, in the MMNI of this study, case studies of mother–child conflicts and their coping strategies were shared with experts, helping participants shift fixed and repetitive parenting attitudes. This intervention process provided theoretical grounds for helping mothers create a stable emotional environment with their children and perform healthy maternal roles by guiding them toward more positive parenting attitudes. It is important to emphasize the expansion of parenting education programs that foster cooperation and empathy in the parent–child relationship, thereby enhancing parenting competence and emotional regulation.

It is necessary to integrate more explicitly the influence of the social environment and the importance of observational learning, which are both core elements of social cognitive theory, into this study. The MMNI was implemented in a group-based format, and the interactions among participants naturally facilitated the development of social support and the modeling of behaviors. This group setting offered participants opportunities to observe and learn from each other’s parenting attitudes and behaviors, which is consistent with the concept of observational learning as outlined in social cognitive theory (19, 23). However, Hypothesis 4, regarding the difference in perceived social support between the experimental and control groups, was rejected. Perceived social support plays a critical role as an environmental factor in enhancing emotional stability and resilience among mothers with ACE (23, 31). Moreover, Perceived social support provides coping strategies in stressful situations and helps overcome difficulties arising in the parenting process (9). Social cognitive theory also suggests that modeling provided within socially supportive environments is essential for positively transforming past experiences learned through exposure to ACE (9, 17). In the MMNI of this study, information about social resource institutions available in the current Korean community was provided, and participants were guided on how to utilize them. Currently, in Korea, various services such as child abuse prevention, early support pilot programs, family function enhancement programs, parenting coaching, and care, medical, and living support services are available. However, the awareness of these services is still low, and access to them is not easy across different residential areas, which limits their utilization (34). Moreover, social support tends to develop and strengthen over time, rather than through short-term interventions; thus, the duration of the intervention in this study may not have been sufficient to capture its full effect. Additionally, the quality and nature of social support are heavily influenced by individuals’ subjective perceptions, and it is possible that the MMNI program was not perceived by participants as a meaningful source of support. Furthermore, if participants were already receiving a certain level of support from family members, the additional impact of the MMNI intervention may have been limited (35). Therefore, future interventions should ensure an adequate duration and incorporate components that address the sub dimensions of social support in both design and evaluation.

Finally, Hypothesis 5, which posited that the parenting behavior of the experimental group would significantly increase compared to the control group, was supported by the results. Parenting behavior refers to the various actions and attitudes parents exhibit to help their children grow and develop, and is correlated with parenting stress, parenting efficacy, mindfulness parenting attitude, and perceived social support (5, 8, 22). Mothers with ACE are more likely to overprotect or, conversely, be avoidant in their parenting, which can lead to emotional behavioral problems in their children, delayed physical growth, social deficits, excessive reliance on smartphones, or behavioral issues (5). In particular, parenting behavior is influenced by personal experiences, learning, and cultural factors. Korea, being a collectivist culture, places importance on obedience to authority and social hierarchy, and as a result, it is often considered inappropriate for mothers to play with their children in non-peer group settings (36). Additionally, due to the emphasis on social harmony in Korean culture, self-expressive behaviors like emotional expression are often suppressed by social norms, and there is a prevalent belief that education is a means of social mobility and success, which emphasizes academic achievement and peer competition (36). These factors influence mothers’ negative parenting behaviors and can further exacerbate negative behaviors in mothers with ACE. Therefore, it is essential to understand the influence of cultural factors on mothers’ parenting behaviors and their reflections on these behaviors (37). The MMNI in this study provided mothers with ACE the opportunity to reflect on their daily routines, emotions, and parenting behaviors, and to share their experiences through a chat room via smartphone, offering them a chance to evaluate their parenting behaviors. This process likely contributed to the significant differences observed in parenting behavior outcomes in this study, supporting the notion that self-reflection influences parenting behavior (25, 31, 32). From the perspective of social cognitive theory, environmental factors play a crucial role in understanding the parenting behaviors of mothers with ACE, as the theory posits that environment is one of the key determinants that both influences and is influenced by individual cognition and behavior (5, 10, 17). Mothers who have experienced ACE often grow up observing negative parenting models, which can adversely affect the way they parent their own children (38). Therefore, the presence of positive social support systems in adulthood, including support from family members, interactions with other parents who are peers, and participation in professional intervention programs, serves as a critical environmental resource that enables these mothers to learn and implement new parenting behaviors (29). In this context, the MMNI provided opportunities for observational learning, allowing mothers with ACE to encounter positive parenting models that differ from those they were exposed to in childhood, and to internalize and apply these new behaviors. Thus, the program may have functioned as a key facilitating factor in promoting positive parenting practices among mothers with ACE.

### Implications

This study emphasizes the significant importance of the MMNI in transforming negative parenting practices into positive ones for mothers who have experienced ACE. Our findings highlight the urgent need for nursing to screen mothers with ACE in clinical and community settings, to recognize and empathize with their past experiences. Furthermore, by investing in the development of nursing intervention programs that transform their negative parenting behaviors, a more compassionate approach to care can be fostered. In addition, raising awareness about ACE and creating a supportive environment for women across the lifespan will contribute to the physical and mental health of mothers. Cultural differences significantly influence individuals’ perceptions and behaviors; therefore, mindfulness-based intervention programs should be adapted to reflect the values and contextual factors of each culture. For mothers in particular, variations in parenting practices, social roles, and sources of stress necessitate culturally sensitive program designs. Such tailored approaches are likely to enhance the effectiveness, acceptance, and sustainability of the interventions. Also, this study’s findings may offer insights into how MMNI can be generalized beyond Korean mothers with ACE to more diverse populations globally. Additionally, the results could be translated into educational modules for healthcare professionals working with individuals who have experienced childhood trauma.

### Limitations

This study has limitations in generalizing the results to all mothers with ACE, as it was conducted using a randomized controlled trial with mothers residing in Seoul and Gyeonggi Province, South Korea. Additionally, considering the COVID-19 pandemic, the virtual intervention program developed and applied in this study may have introduced uncontrollable variables. Therefore, further research is needed to investigate the long-term effects of MMNI. Although this study demonstrated positive short-term outcomes, future research should investigate whether these effects lead to sustained changes in parenting behaviors and child development over time. For example, follow-up studies using repeated measures ANOVA or ANCOVA could be conducted to examine long-term effects. This study relies on self-reported data, passive delivery of information, brief intervention duration, and lack of interpersonal engagement, which may be affected by cultural desirability bias or subjective interpretation, leading to inaccuracies. Future research could integrate additional objective measurements or observational data to strengthen the validity of the results.

## Conclusion

This study aimed to develop and apply the MMNI for mothers with ACE in Korea and to verify its effectiveness through an RCT. The results showed significant differences in parenting stress, parenting efficacy, mindfulness parenting attitude, and parenting behavior in the experimental group, supporting the hypotheses. However, perceived social support did not show significant differences. This study contributes scientifically by providing evidence that a structured, MMNI can enhance parenting attitudes, increase parenting efficacy, and promote mindfulness parenting behavior among mothers with ACE. The program facilitated improved emotional regulation and reflective caregiving behaviors, supporting healthier mother–child interactions. It offers a trauma-informed framework for parenting support, addressing intergenerational impacts of ACE.

## Data Availability

The original contributions presented in the study are included in the article/supplementary material, further inquiries can be directed to the corresponding author.

## Footnotes

^1^ https://cris.nih.go.kr/cris/search/detailSearch.do?seq=21959&search_page=L

## Author’s note

This article is a revision of the first author’s doctoral dissertation from Chung-Ang University.
